# Inter- and intra-combinatorial regulation by transcription factors and microRNAs

**DOI:** 10.1186/1471-2164-8-396

**Published:** 2007-10-30

**Authors:** Yiming Zhou, John Ferguson, Joseph T Chang, Yuval Kluger

**Affiliations:** 1 Department of Cell Biology, New York University School of Medicine, New York, New York 10016, USA; 2 Department of Statistics, Yale University, New Haven, CT 06520, USA

## Abstract

**Background:**

MicroRNAs (miRNAs) are a novel class of non-coding small RNAs. In mammalian cells, miRNAs repress the translation of messenger RNAs (mRNAs) or degrade mRNAs. miRNAs play important roles in development and differentiation, and they are also implicated in aging, and oncogenesis. Predictions of targets of miRNAs suggest that they may regulate more than one-third of all genes. The overall functions of mammalian miRNAs remain unclear. Combinatorial regulation by transcription factors alone or miRNAs alone offers a wide range of regulatory programs. However, joining transcriptional and post-transcriptional regulatory mechanisms enables higher complexity regulatory programs that in turn could give cells evolutionary advantages. Investigating coordinated regulation of genes by miRNAs and transcription factors (TFs) from a statistical standpoint is a first step that may elucidate some of their roles in various biological processes.

**Results:**

Here, we studied the nature and scope of coordination among regulators from the transcriptional and miRNA regulatory layers in the human genome. Our findings are based on genome wide statistical assessment of regulatory associations ("interactions") among the sets of predicted targets of miRNAs and sets of putative targets of transcription factors. We found that combinatorial regulation by transcription factor pairs and miRNA pairs is much more abundant than the combinatorial regulation by TF-miRNA pairs. In addition, many of the strongly interacting TF-miRNA pairs involve a subset of master TF regulators that co-regulate genes in coordination with almost any miRNA. Application of standard measures for evaluating the degree of interaction between pairs of regulators show that strongly interacting TF-miRNA, TF-TF or miRNA-miRNA pairs tend to include TFs or miRNAs that regulate very large numbers of genes. To correct for this potential bias we introduced an additional Bayesian measure that incorporates not only how significant an interaction is but also how strong it is. Putative pairs of regulators selected by this procedure are more likely to have biological coordination. Importantly, we found that the probability of a TF-miRNA pair forming feed forward loops with its common target genes (where the miRNA simultaneously suppresses the TF and many of its targets) is increased for strongly interacting TF-miRNA pairs.

**Conclusion:**

Genes are more likely to be co-regulated by pairs of TFs or pairs of miRNAs than by pairs of TF-miRNA, perhaps due to higher probability of evolutionary duplication events of shorter DNA sequences. Nevertheless, many gene sets are reciprocally regulated by strongly interacting pairs of TF-miRNA, which suggests an effective mechanism to suppress functionally related proteins. Moreover, the particular type of feed forward loop (with two opposing modes where the TF activates its target genes or the miRNA simultaneously suppresses this TF and the TF-miRNA joint target genes) is more prevalent among strongly interacting TF-miRNA pairs. This may be attributed to a process that prevents waste of cellular resources or a mechanism to accelerate mRNA degradation.

## Background

MiRNAs belong to a class of non-coding small RNAs. The first miRNA was found by Victor Ambros and his colleagues [1,2]. Its mature sequence contains only 21~24 nucleotides. Lee et al. and Wightman et al. [2,3] first found that miRNAs might regulate protein expression at a post-transcriptional stage. The properties of this novel and vital class of regulators are being extensively studied. Although some miRNAs may be transcribed by RNA polymerase III (Pol III) [4], it is believed that most miRNAs are transcribed by RNA polymerase II (Pol II) [5]. In mammalian cells, the original transcripts are cleaved by the Drosha RNase II endonuclease into 60~70nt and then exported to cytoplasm by Exportin-5 and its cofactor Ran-GTP [6-9]. Finally, Dicer crops the exported miRNAs in the cytoplasm into 21nt mature miRNAs [10-12]. Mature miRNAs are eventually transferred to Argonaute proteins and serve as guides in mRNA silencing [13]. Expression studies have shown that many miRNAs have tissue-specific or developmental-stage-specific expression patterns [5,14]. Emerging *in vivo *and *in vitro *experiments are showing that miRNAs regulate a broad diversity of cellular processes, including differentiation, development, aging, apoptosis, oncogenesis and metabolism [5,15-19].

Identification of targets of miRNAs is a critical step in deciphering the function of miRNAs. Comprehensive understanding of the underlying mechanisms of miRNA binding is lacking. Efficient high-throughput experimental methods for miRNA target identification are still underdeveloped. Therefore, genome-wide identification of miRNA targets is currently based on computational predictive models. In plants, these predictions are straightforward since a plant gene typically contains a sequence that is complementary to the sequence of the whole miRNA [20]. Moreover, most targets of miRNAs in plants are transcription factors [5,20]. In metazoan, the situation is more complex since a perfect complementarity is not necessary for a miRNA to recognize its targets. Recently developed algorithms to predict the targets of metazoan miRNAs include PicTar [21], miRanda [22] and TargetScan [23]. These algorithms employ similar sets of rules of the form: 1) the ~7nt core region at the 5' of miRNAs should approximately match with the 3' untranslated regions (UTR) of the putative target genes; 2) the free energy of the entire miRNA/mRNA duplex should be below a cutoff value; 3) the binding sites should be conserved among several different species. Small differences in the implementation of the rules of these algorithms contribute to discrepancies among their predicted targets. Despite the lack of sufficient numbers of experimentally verified targets for accurate assessment of the overall sensitivity and specificity of the predictions obtained by these algorithms, recent reports indicate that a large class of miRNA targets can be confidently predicted [24,25].

Studying the extent to which miRNAs interact in a combinatorial fashion with other regulators (e.g. TFs and signal molecules) and among themselves is an important step for further elucidating the functions of miRNAs at a system-wide level. Earlier studies have demonstrated how combinatorial transcriptional regulation affects expression patterns across a variety of biological conditions [26,27]. Recently, Cui et al. employed a statistical approach to decipher global relationships in miRNA regulation, cellular signaling networks [28] and predicted transcription regulatory networks [29]. In these studies, Cui et al. examined the relationship between the centrality (number of connections) of each node (gene) in signaling networks or transcription regulatory networks and abundance of miRNA binding. They found that miRNAs typically target: a) positive regulatory motifs (three or four proteins which positively regulate each other); b) downstream network components and the highly connected scaffolds in a signalling network; and c) genes whose promoter regions include a large number of putative transcription factor binding sites.

Here, we derived a combined draft of the human TF and miRNA regulatory network and investigated the extent of combinatorial regulation within and between the transcriptional and miRNA regulatory layers. To determine biologically coordinated regulation by any pair of TF or miRNA regulators, we utilized several measures for assessing the statistical interaction between the miRNA and TF variables. When we assess interaction strength by employing interaction estimators such as the correlation between the binding profiles of each pair of regulators, Fisher's exact test, the Chi Square test or similar tests for association, we are more likely to identify pairs of strongly interacting regulators for which at least one of the two regulators targets a large number of genes. To reveal biological coordination between regulators that target a smaller number of genes, we utilized an additional non-standard interaction measure based on a Bayesian approach.

## Results

### Intra and inter-coordinated gene regulation by pairs of miRNAs and TFs

To identify groups of genes that are likely to be combinatorially regulated by TF-TF, miRNA-miRNA and TF-miRNA pairs, we constructed a combined transcriptional and miRNA putative regulatory network using the TRANSFAC transcription factor database [30] and the PicTar miRNA database [21]. The PicTar database stores the predicted targets of 168 distinct human miRNAs. To obtain an analogous list for the predicted targets of the 236 human transcription factors whose probability weight matrices (PWMs) are stored in the Transfac(R) 9.4 database, we scanned the promoters of all annotated transcripts and matched them against each of these PWMs. To assess the robustness of our general observations, we varied the parameters used in constructing the predictive links of the transcriptional binding network (see details in the Methods section above). To label the predicted targets of each TF in the transcription regulatory network, we used the Refseq database whose identifiers are uniquely associated with gene transcription start sites and their corresponding promoters. Use of the Refseq database enabled us to combine the transcriptional network with the PicTar database that provides annotation of the miRNA targets in terms of Refseq IDs. We then linked each Refseq ID to its corresponding gene symbol using the NCBI gene database. A gene that was mapped to multiple Refseq IDs, due to alternative splicing, was marked as a target of a miRNA or TF if the latter regulates at least one of the gene's Refseq transcripts. With this in mind, we will present the results and discussion in term of gene symbols instead of RefSeq IDs.

It is plausible that the degree of coordinated gene co-regulation by pairs of TFs, miRNAs or TF-miRNA regulators can be inferred by utilizing statistical association (interaction) measures for quantifying the significance and size of the overlap between the sets of predicted targets of each pair of regulators. To evaluate the significance of the overlap, viz., the extent to which the targets of one regulator are enriched in targets of another regulator, we first applied Fisher's Exact Test. We then transformed the p-values obtained from Fisher's Exact Test into q-values (as a False Discovery Rate Correction) [31].

**Figure 1:**
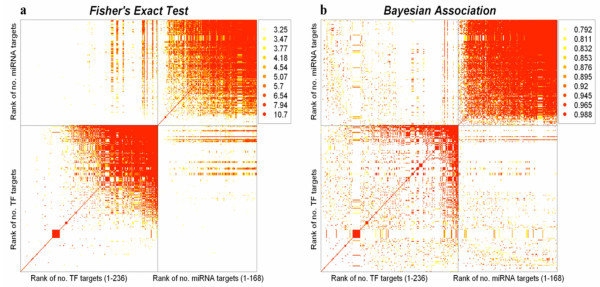
**TF and miRNA interaction heatmap**. Each pixel on Figures 1a and 1b represents the association of a unique pair of regulators. Figure 1a measures this association by a Fisher's Exact Test p-value (dark pixels represent lower p-values or alternatively a higher value of -log_10_*p*). Figure 1b measures the association by the Bayesian probability Pr{logOR>0.6} (Here a dark pixel means a high probability). The TFs and miRNAs are ordered so that the number of targets of each regulator increases as one moves across the Figure from left to right on the horizontal axis, and also up the vertical axis. Both Figures 1a and 1b illustrate that while TF-TF and miRNA-miRNA associations are common, TF-miRNA interactions are less so. The TF-miRNA rectangles of Fig 1a demonstrate that the most significant associations (as found by Fisher's Exact Test) tend to involve TF-miRNA pairs with the TF having a large number of targets. In the corresponding areas of Figure 1b, we see a more uniform sprinkling of dark points, indicating that the Bayesian approach is less sensitive to sample size effects. The stripes on the TF-miRNA rectangles of both figures demonstrate that certain TFs are associated with almost all the miRNAs – while, surprisingly, many TFs with a similar number of targets seem to not be significantly associated with any miRNA.

We found that co-regulation by TF pairs or miRNA pairs is substantially more abundant compared to co-regulation by TF-miRNA pairs. This trend is demonstrated by the block diagonal structure of the (ranked-based) scatter plot of the significant regulatory pairs, as shown in Fig. 1a. We found that about 5.5% (2212 out of 39648) of the TF-miRNA pairs share a significant number of targets (q-value < 0.01). These 2212 pairs include 85 TFs and 155 miRNAs. Inspection of the off-diagonal TF-miRNA blocks in Fig. 1a show that only one third of the TFs share a significant number of targets with at least one miRNA, while a majority of miRNAs have significant overlap with targets of one or more TFs. Moreover, the red stripes in the off-diagonal blocks shown in Fig. 1a, indicate that a few master TF regulators (TFs with a very large number of targets) co-regulate genes in coordination with a large number of miRNAs. This suggests that regulation by some TFs is accompanied by additional "corrections" or fine-tuning of protein concentrations via combinatorial regulation with multiple miRNAs. As seen in Figure 1a, TFs with a larger number of targets are more likely to have significant overlap (q-value < 0.01) with targets of miRNAs. However, this is most probably due to a sample-size effect associated with p-values: when testing multiple hypotheses in a situation where the sample sizes differ with each test, the statistical power will generally be higher for tests run on larger samples. In testing TF-miRNA associations, a proxy for sample size is the number of target genes associated with the TF and miRNA. There may also be important TF-miRNA associations where each regulator in the TF-miRNA pair has only a small number of targets, however, the power of Fisher's Exact Test may be too low for us to detect such pairs. In addition, a significant p-value or q-value for a TF-miRNA pair gives us no guarantee that the association is of practical importance: small and uninteresting effect sizes can still show small p-values if the sample size is large enough.

**Figure 2:**
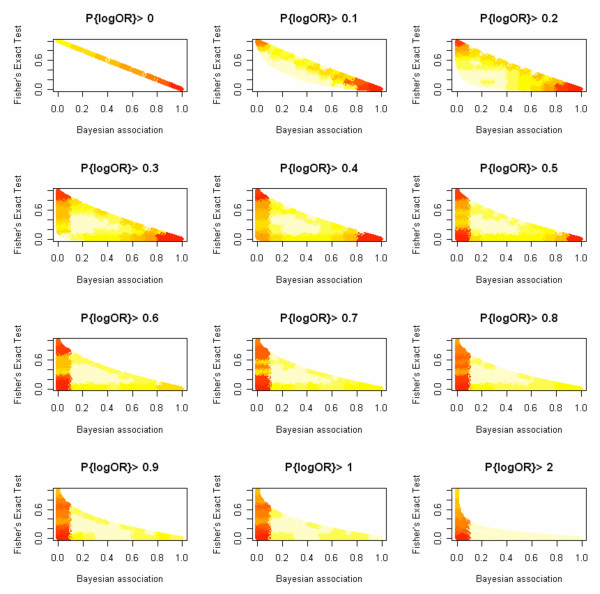
**Comparison of Fisher's exact test and Bayesian association score**. Scatter plots of Fisher's Exact Test p-value as a function of Bayesian association score. The 2D distributions demonstrate how the relationship between Fisher's Exact Test and our Bayesian score depends on the logOR threshold we use. Each sub-plot represents a different threshold value ranging from 0 to 1 – as indicated by each subtitle. For a particular threshold value, a pixel on the plot represents the local density of miRNA-TF pairs having the corresponding p-value (from the y-axis) and Bayesian Probability (from the x-axis). Here darker shaded regions indicate higher densities. For a 0 threshold, the Bayesian Test and Fisher's Test agree exactly. As we increase the threshold, we see fewer and fewer TF-miRNA pairs that are highly associated as measured by both ranking criteria (pairs whose measures approach 1 at the x-axis and 0 at the y-axis). The higher the threshold, the more emphasis we are placing on the size of the TF-miRNA association (as measured by a log Odds Ratio) and the less emphasis on sample size. Note that a very high Bayesian probability implies that the associated p-value will be small, no matter what threshold we use.

With these issues in mind, we used an alternative approach to identify associated miRNA-TF pairs. Our new approach allows us to identify additional associated miRNA-TF pairs such that the TF and the miRNA do not necessarily have a large number of targets. It also gives greater emphasis than Fisher's Exact Test to the effect size of the TF-miRNA association. With this method, we rank by how sure we are that the TF-miRNA association, measured by the log Odds Ratio (logOR), exceeds a certain threshold. More formally, we compare the various TF-miRNA associations by examining the Bayesian posterior probability Prob{logOR >c} for each pair. The threshold *c *is chosen from the data. Larger values of this statistic imply a greater level of association between the TF and miRNA. Indeed, as can be seen from Fig. 2, no matter how we choose *c*, a large value of Prob{logOR >c} implies the corresponding Fisher's Test p-value will be small. As demonstrated in Fig. 1b, in contrast to Fisher's test, the most salient TF-miRNA associations according to our Bayesian methodology do not necessarily involve TF-miRNA pairs where both regulators have many targets.

### Co-regulation of related genes

The statistical relationships reported in the previous section are based on predictive input of the miRNA targets and a mix of predictive and experimental input of the TF targets. We obtain similar conclusions using other databases of miRNA targets [23]. The common presumption among genomics experimentalists is that the overall false positive rate in predicting TF or miRNA binding sites using various prediction methods is in the range of 50%-80%. It is plausible that the quality of these predictions can be improved by reproducing the genome-wide analyses of the previous section for a smaller set of functionally related genes.

In the current study, we examined the degree to which gene sets regulated by any TF, miRNA or pair of TF-miRNA intersect with thousands of functionally related sets of genes annotated in the biological process classification Gene Ontology (GO) catalogue [32]. Using Fisher's Exact Test, we found that out of the 2212 strongly interacting TF-miRNA pairs (OMTs, q-value<0.01), 1223 are significantly enriched in at least one GO term. These 1223 OMTs involve 48 TFs and 121 miRNAs and are enriched in 96 GO terms, while the targets of 117 of 168 miRNAs and the targets of 105 of 236 TFs are enriched in 71 and 206 GO terms, respectively.

Interestingly, we found that gene sets associated with cell adhesion related GO terms including homophilic cell adhesion, cell-cell adhesion and the less specific cell adhesion category, are enriched by 40, 25 and 14 miRNAs, respectively, but not enriched by any TF. The groups of gene targets bound by the 14 miRNAs enriched in the less specific cell adhesion category are a subset of the groups bound by the 25 miRNAs enriched in cell-cell adhesion activity.

Similarly, the 25 groups associated with cell-cell adhesion are a subset of the 40 miRNAs gene sets enriched by homophilic cell adhesion. This observation is not only consistent with the current presumption that miRNAs are only found in multi-cellular species, but provides an important clue for their cellular roles and origin. Moreover, since the reduction in cell adhesion correlates with tumor invasion, it implicates these miRNAs with cancer metastasis. We found that 8 out of the 40 cell adhesion enriched miRNAs are present in a dataset of 21 miRNAs that are up or down regulated in a variety of cancers [33].

### Feed forward loops

**Figure 3:**
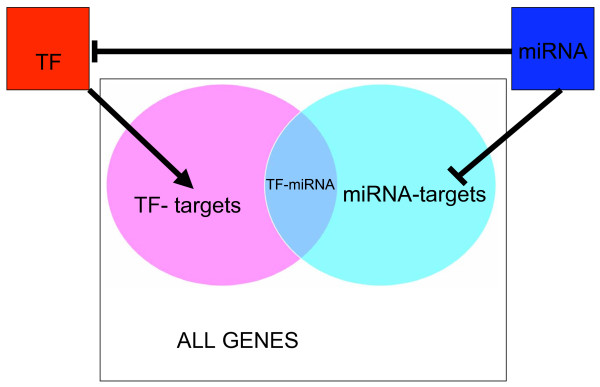
**Feed Forward Loop (FFL)**. A feed forward loop (FFL) is a regulatory motif in which regulator A regulates another regulator denoted by B, and both regulators A and B regulate a common target C.

There are two possible mechanisms by which miRNAs repress mRNA translation: 1) blocking mRNA translation without degrading the mRNA targets; 2) direct degradation of mRNA targets by miRNA. Both mechanisms allow a rapid reduction in the number of translated proteins compared to the long half-life time required to suppress the mRNAs via transcriptional regulation. Predictively, miRNAs could repress hundreds of mRNAs, which in turn could be wastefully produced if the relevant TFs do not change their level of activity. To avoid this inefficiency and accelerate mRNA degradation, we hypothesize that a TF that shares a large number of targets with a miRNA is more likely be the target of this miRNA. This is a feed forward mechanism in which a gene target and its TF regulator are simultaneously suppressed by the same miRNA and the gene is activated by the TF once the miRNA levels are down regulated (See Fig. 3).

We compared the abundance of feed forward loops in the group of strongly interacting TF-miRNA pairs (Fisher's Exact Test q-value < 0.01) to their abundance in the remaining TF-miRNA pairs. Using logistic regression (to control for variation in the number of miRNA targets), we found that the proportion of feed forward loops among the strongly interacting TF-miRNA pairs is significantly higher (p~1.86 × 10^-9^) than among the remaining pairs.

**Figure 4:**
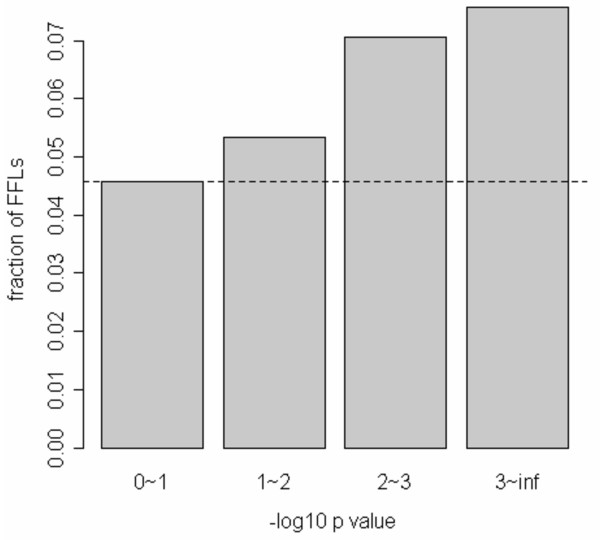
**Relationship between FFL and TF/miRNA association**. Fraction of FFLs as a function of the statistical significance for TFs and miRNAs association. The histogram displays the fraction of FFLs that result in each bin, when grouping miRNA/TF/GO triplets according to their log p-value of joint-association. To generate this histogram, we used a slightly restricted set of biological-process GO terms, such that each group includes at least one gene that is a predicted target of a TF and a miRNA. The plot suggests that when a miRNA/TF/GO triplet is significantly associated, the corresponding miRNA and TF are more likely to form a feed forward loop.

It is plausible that the effect of false predictions of TF and miRNA targets on the overall conclusions may be reduced by evaluating the strength of the interaction of each TF-miRNA pair within a GO functional context. With this in mind, we examined the abundance of FFLs as a function of the extent (significance) to which triplets of gene sets associated with a TF, a miRNA and a GO term overlap with each other. We evaluated the significance of the overlap of each triplet using a constrained sum involving a product of two hypergeometric distributions. The first distribution quantifies the significance of the intersection between the targets of a TF with those of a miRNA, and the second one quantifies the overlap between this intersection and a gene set associated with a GO term (see methods section for details). As shown in Fig. 4, the smaller the p-value, the higher the fraction of FFLs among all the triplets associated with the corresponding p-value.

We note that our database contains all the predicted FFLs. FFLs that are not associated with strongly interacting TF-miRNA pairs could also provide useful information in exploring the regulation of genes of interest. For example, hsa-miR-29b and its predicted MYBL2 target are deregulated in breast cancer [33,34]. This miRNA and its target gene form putative FFLs with BACH2 and SP1. Another example relevant for breast cancer is the hsa-miR-17-5p/EREG pair of miRNA and predicted target gene that putatively form FFLs with E2F1, EGR2, FOXJ2 and RUNX1.

## Discussion and conclusions

In this work we integrated transcriptional and post-transcriptional putative human gene regulatory networks and studied the landscape of coordinated gene regulation within and between these two regulatory layers.

We determined the degree of combinatorial regulation between pairs of regulators using statistical measures that quantify the level of association between the sets of targets of each of these pairs. We first used standard association measures that employ a hypothesis testing approach to determine the significance of the interaction between pairs of regulators.

We then introduced an additional novel Bayesian approach that allows us to simultaneously assess the significance and strength of the interactions between the regulators. Since significance-based measures are strongly dependent on sample size (the number of targets of each regulator), the use of our new measure allows us to identify a balanced list of top interacting pairs of regulators that takes into consideration not only sample size but also effect size.

Our results suggest that genes are more likely to be co-regulated by pairs of TFs or pairs of miRNAs than by pairs of TF-miRNA. One possible explanation for this observation is that evolutionary duplication events of shorter DNA sequences are more likely. This implies that binding sites of regulators of a given genome whose distance along the DNA is short have a higher probability to be duplicated in a similar configuration in one or more positions in descendent genomes. Remarkably, we found that the frequency of co-appearance of pairs of 7-mer core sequences, associated with known miRNAs is higher in the promoter regions than their co-appearance at large genomic distance where one member of the pair is positioned in the 3'UTR region and the other in the promoter region (data not shown). This is not obvious due to the fact that the prevalence of these sequences in non 3'UTR regions is low. This result is consistent with our suggestion that short segments of DNA are more likely to be duplicated than longer segments.

Regulation by pairs of TFs or miRNAs is more common than by TF-miRNA pairs. However, many gene sets are still reciprocally regulated by strongly interacting pairs of TF-miRNA, which suggests an efficient mechanism to suppress functionally related proteins. In addition, the higher prevalence of feed forward loops (FFL) among strongly interacting TF-miRNA pairs, in which the miRNA simultaneously suppresses the TF and many of its targets may be attributed to a mechanism designed to prevent waste of cellular resources by prohibiting two simultaneous contradictory processes: one in which the binding of the TF to the promoter of the target gene stimulates production of additional mRNA copies of this gene and the other where the miRNA degrades these copies.

While there are many experimental results about feedback loops involving miRNAs [35-40], the role of FFLs involving miRNAs is much less explored. Previous experiments, studying the operational mechanics of FFLs focused on specific biological pathways, such as metabolic pathways or signal transduction pathways, or involved the interaction of TFs alone (without the input of miRNAs). Recently, O'Donnell et al. discovered a FFL in which c-Myc activates E2F1, miR17-5p and miR-20 and the two miRNAs in turn repress E2F1 [41]. Since E2F1 is also an activator of c-Myc, the FFL provides a mechanism through which c-Myc simultaneously activates E2F1 transcription and limits its translation, allowing a tightly controlled proliferative signal. Three recent studies suggest that the architecture of a FFL of this type (where the miRNA is regulated by a TF and both of these regulators regulate another TF) maybe associated with buffering of the noise in the environment of the FFL system [42-44]. The configuration of FFL investigated here involves two inhibitory links emanating from the regulator in the top of the FFL and one stimulatory link from the other regulator of this FFL. Mangan and Alon [45] found that out of the eight possible FFL configurations (where each of the FFL links is either inhibitory or stimulatory) this configuration is the second most common FFL in the yeast transcriptional network. One advantage of this kind of FFL is that at steady state it allows the regulation of its target gene by stimuli acting on any of its two regulators.

A FFL composed of post-transcriptional miRNA and transcriptional TF regulators possesses an additional property. Since miRNAs directly repress mRNAs, this FFL system has a shorter response time to a stimulatory signal compared to the purely transcriptional FFLs that are discussed in [45]. It is also worth mentioning that once the system reaches a steady state, in which the miRNA is at high level and both the TF and target are at low levels, a transient disturbance in the signal that controls the miRNA level will only have a small effect on the state of the system. This 'denoising' function of miRNA is getting supports from experiments [18,46]. Thus, it is plausible that such FFLs with a miRNA at the origin offer an alternative mechanism to dampen excessive fluctuations in the mRNA level of a target gene. We infer the denoising property in this type of FFL architecture based on the distinct expected response times to increase and decrease mRNA concentration levels. On one hand a short time is needed to dampen the mRNA levels of the target gene and the TF following the onset of miRNA. On the other hand once the miRNA is turned off, the return to a state where the concentration of mRNA of the target gene is higher is gradual, since the TF first has to be transcribed, translated and transported to the nucleus, whereupon it can bind to the promoter region of the gene and hence increase its mRNA level.

Previous studies [47,48] suggested that miRNAs might fine-tune the expression level of single genes by repressing their translation. In this study we provide a genome-wide picture revealing the scope of combinatorial regulation by pairs of TF-miRNA whose role may be associated not only with a fine-tuning mechanism of the transcriptional network, but also with a 'quick-OFF-slow-ON' switching devise as well as a machinery designed to preserve resources. Our analyses also allude to additional potential unknown roles for miRNAs, e.g. regulating multiple cellular processes such as cell-cell adhesion.

## Methods

### Construction of the TF binding network

The results presented in this study are based on one choice of parameters used to derive a predictive binding transcriptional network. To validate the robustness of our general observations, we constructed several predictive transcriptional binding networks by exploring the parameter space. Specifically, we used different scanning ranges of promoter regions (1000 bp or 2000 bp upstream of the transcription start site (TSS) to 200bp, 1000bp, 2000bp or position of the second exon downstream of the TSS), and several TRANSFAC thresholds for determining a protein-DNA binding site, including the requirement that there is a sufficient match between the TF PWM in the homologous gene in mouse. We also used only the promoter regions that are conserved in both human and mouse. The results reported here are based on networks constructed using existing experimental binding data and predicted bindings that were generated by using sequences 2000 bp upstream and 2000 bp downstream of RefSeq identified transcription start sites and cut-offs intended to minimize false positives, as provided by TRANSFAC.

The human and mouse Refseq IDs were extracted from the most recently updated human and mouse genome builds (NCBI Build 36.1 and NCBI Build 36 respectively). To define direct regulation connectivity between a transcription factor (TF) and a gene, we used the default parameters (minimization of false positives) for matching the position weight matrices (PWM) stored in Transfac^® ^(version 9.4) with the promoter regions by using the Transfac^® ^MATCH algorithm [49]. TF-target gene links found in the matching step were retained only if the PWM match with the human promoter region, as well as with the promoter of the orthologous gene in the mouse genome (found in the HomoloGene database [50]), were above the default cutoff.

### Predicted targets of miRNAs

Predicted targets of miRNAs were assembled from the PicTar Database [21]. In this study we used the predicted targets, which are conserved in human, chimpanzee, mouse, rat, and dog genomes.

### GO annotation

The annotations of Gene Ontology (GO) were downloaded from [51]. We mapped the GO terms to NCBI Gene IDs using the index file from [52].

### Enrichment analysis

P-values representing TF-miRNA enrichment were calculated using Fisher's Exact Test. For a TF-miRNA pair such that the TF targets *m *genes (out of a possible *N*) and the miRNA targets *n *genes, the p-value for TF-miRNA association is given by

p=∑i=kmin⁡(n,m)(mi)(N−mn−i)(Nn)
 MathType@MTEF@5@5@+=feaafiart1ev1aaatCvAUfKttLearuWrP9MDH5MBPbIqV92AaeXatLxBI9gBaebbnrfifHhDYfgasaacH8akY=wiFfYdH8Gipec8Eeeu0xXdbba9frFj0=OqFfea0dXdd9vqai=hGuQ8kuc9pgc9s8qqaq=dirpe0xb9q8qiLsFr0=vr0=vr0dc8meaabaqaciaacaGaaeqabaqabeGadaaakeaacqWGWbaCcqGH9aqpdaaeWbqaamaalaaabaWaaeWaaeaafaqabeGabaaabaGaemyBa0gabaGaemyAaKgaaaGaayjkaiaawMcaamaabmaabaqbaeqabiqaaaqaaiabd6eaojabgkHiTiabd2gaTbqaaiabd6gaUjabgkHiTiabdMgaPbaaaiaawIcacaGLPaaaaeaadaqadaqaauaabeqaceaaaeaacqWGobGtaeaacqWGUbGBaaaacaGLOaGaayzkaaaaaaWcbaGaemyAaKMaeyypa0Jaem4AaSgabaGagiyBa0MaeiyAaKMaeiOBa4MaeiikaGIaemOBa4MaeiilaWIaemyBa0MaeiykaKcaniabggHiLdaaaa@4FD5@

where *k *is the number of genes targeted by both the TF and the miRNA.

We implemented a False Discovery Rate correction by transforming the Fisher's Exact Test p-values into q-values [31]. We used the Statistics program R and associated "qvalue" package to perform these calculations.

We propose a new test extending Fisher's Exact Test to a situation in which we wish to test for a 3-group association. Given a set of *N *genes and three subsets of these genes of sizes *n*, *m *and *o*, the *p*-value of a joint association between all three subsets is calculated as:

p=∑i=kmin⁡(n,m)∑j=kmin⁡(i,o)(mi)(N−mn−i)(Nn)×(ij)(N−io−j)(No)
 MathType@MTEF@5@5@+=feaafiart1ev1aaatCvAUfKttLearuWrP9MDH5MBPbIqV92AaeXatLxBI9gBaebbnrfifHhDYfgasaacH8akY=wiFfYdH8Gipec8Eeeu0xXdbba9frFj0=OqFfea0dXdd9vqai=hGuQ8kuc9pgc9s8qqaq=dirpe0xb9q8qiLsFr0=vr0=vr0dc8meaabaqaciaacaGaaeqabaqabeGadaaakeaacqWGWbaCcqGH9aqpdaaeWbqaamaaqahabaWaaSaaaeaadaqadaqaauaabeqaceaaaeaacqWGTbqBaeaacqWGPbqAaaaacaGLOaGaayzkaaWaaeWaaeaafaqabeGabaaabaGaemOta4KaeyOeI0IaemyBa0gabaGaemOBa4MaeyOeI0IaemyAaKgaaaGaayjkaiaawMcaaaqaamaabmaabaqbaeqabiqaaaqaaiabd6eaobqaaiabd6gaUbaaaiaawIcacaGLPaaaaaGaey41aq7aaSaaaeaadaqadaqaauaabeqaceaaaeaacqWGPbqAaeaacqWGQbGAaaaacaGLOaGaayzkaaWaaeWaaeaafaqabeGabaaabaGaemOta4KaeyOeI0IaemyAaKgabaGaem4Ba8MaeyOeI0IaemOAaOgaaaGaayjkaiaawMcaaaqaamaabmaabaqbaeqabiqaaaqaaiabd6eaobqaaiabd+gaVbaaaiaawIcacaGLPaaaaaaaleaacqWGQbGAcqGH9aqpcqWGRbWAaeaacyGGTbqBcqGGPbqAcqGGUbGBcqGGOaakcqWGPbqAcqGGSaalcqWGVbWBcqGGPaqka0GaeyyeIuoaaSqaaiabdMgaPjabg2da9iabdUgaRbqaaiGbc2gaTjabcMgaPjabc6gaUjabcIcaOiabd6gaUjabcYcaSiabd2gaTjabcMcaPaqdcqGHris5aaaa@729A@

where *k *is the overlap between the three groups.

### Bayesian Methodology

Our likelihood model is based upon the multinomial distribution, which is a common choice in modelling count data. For each TF-miRNA pair, suppose that we partition the set of all genes into four subsets according to whether each gene is a target of the TF or miRNA and count the number of genes in each subset: *N*_11_, *N*_12_, *N*_21 _and *N*_22 _(e.g. *N*_11 _is the number of genes targeted by both the TF and the miRNA and *N*_12 _is the number of genes targeted by the TF but not by the miRNA). We regard each model parameter *p*_*ij *_as the probability that a gene contributes to the count *N*_*ij *_(which we assume is constant for all genes). Under the assumption that the genes are independent with respect to whether or not they are targets of each regulator, the likelihood equation is:

L(p11,p12,p21,p22)∝p11N11p12N12p21N21p22N22
 MathType@MTEF@5@5@+=feaafiart1ev1aaatCvAUfKttLearuWrP9MDH5MBPbIqV92AaeXatLxBI9gBaebbnrfifHhDYfgasaacH8akY=wiFfYdH8Gipec8Eeeu0xXdbba9frFj0=OqFfea0dXdd9vqai=hGuQ8kuc9pgc9s8qqaq=dirpe0xb9q8qiLsFr0=vr0=vr0dc8meaabaqaciaacaGaaeqabaqabeGadaaakeaacqWGmbatcqGGOaakcqWGWbaCdaWgaaWcbaGaeGymaeJaeGymaedabeaakiabcYcaSiabdchaWnaaBaaaleaacqaIXaqmcqaIYaGmaeqaaOGaeiilaWIaemiCaa3aaSbaaSqaaiabikdaYiabigdaXaqabaGccqGGSaalcqWGWbaCdaWgaaWcbaGaeGOmaiJaeGOmaidabeaakiabcMcaPiabg2Hi1kabdchaWnaaDaaaleaacqaIXaqmcqaIXaqmaeaacqWGobGtdaWgaaadbaGaeGymaeJaeGymaedabeaaaaGccqWGWbaCdaqhaaWcbaGaeGymaeJaeGOmaidabaGaemOta40aaSbaaWqaaiabigdaXiabikdaYaqabaaaaOGaemiCaa3aa0baaSqaaiabikdaYiabigdaXaqaaiabd6eaonaaBaaameaacqaIYaGmcqaIXaqmaeqaaaaakiabdchaWnaaDaaaleaacqaIYaGmcqaIYaGmaeaacqWGobGtdaWgaaadbaGaeGOmaiJaeGOmaidabeaaaaaaaa@5C71@

for *p*_*ij *_≥ 0 and ∑_*ij *_*p*_*ij *_= 1. The Dirichlet prior distribution we are using has the form: *f*(*p*_11_, *p*_12_, *p*_21_, *p*_22_) ∝ *p*_11 _^*α *- 1 ^*p*_22 _^*α *- 1 ^[53] and taking the product of the likelihood and prior, we obtain : g(p11,p12,p21,p22)∝p11N11+α−1p12N12p21N21p22N22+α−1
 MathType@MTEF@5@5@+=feaafiart1ev1aaatCvAUfKttLearuWrP9MDH5MBPbIqV92AaeXatLxBI9gBaebbnrfifHhDYfgasaacH8akY=wiFfYdH8Gipec8Eeeu0xXdbba9frFj0=OqFfea0dXdd9vqai=hGuQ8kuc9pgc9s8qqaq=dirpe0xb9q8qiLsFr0=vr0=vr0dc8meaabaqaciaacaGaaeqabaqabeGadaaakeaacqWGNbWzcqGGOaakcqWGWbaCdaWgaaWcbaGaeGymaeJaeGymaedabeaakiabcYcaSiabdchaWnaaBaaaleaacqaIXaqmcqaIYaGmaeqaaOGaeiilaWIaemiCaa3aaSbaaSqaaiabikdaYiabigdaXaqabaGccqGGSaalcqWGWbaCdaWgaaWcbaGaeGOmaiJaeGOmaidabeaakiabcMcaPiabg2Hi1kabdchaWnaaDaaaleaacqaIXaqmcqaIXaqmaeaacqWGobGtdaWgaaadbaGaeGymaeJaeGymaedabeaaliabgUcaRGGaciab=f7aHjabgkHiTiabigdaXaaakiabdchaWnaaDaaaleaacqaIXaqmcqaIYaGmaeaacqWGobGtdaWgaaadbaGaeGymaeJaeGOmaidabeaaaaGccqWGWbaCdaqhaaWcbaGaeGOmaiJaeGymaedabaGaemOta40aaSbaaWqaaiabikdaYiabigdaXaqabaaaaOGaemiCaa3aa0baaSqaaiabikdaYiabikdaYaqaaiabd6eaonaaBaaameaacqaIYaGmcqaIYaGmaeqaaSGaey4kaSIae8xSdeMaeyOeI0IaeGymaedaaaaa@657B@ as our posterior distribution. As an alternative to Fisher's Exact Test, we use the statistic:

P{log⁡OR>c}=∫pij≥0,∑ijpij=1I{log⁡(p11p22p12p21)>c}g(p11,p12,p21,p22)Πijdpij,
 MathType@MTEF@5@5@+=feaafiart1ev1aaatCvAUfKttLearuWrP9MDH5MBPbIqV92AaeXatLxBI9gBaebbnrfifHhDYfgasaacH8akY=wiFfYdH8Gipec8Eeeu0xXdbba9frFj0=OqFfea0dXdd9vqai=hGuQ8kuc9pgc9s8qqaq=dirpe0xb9q8qiLsFr0=vr0=vr0dc8meaabaqaciaacaGaaeqabaqabeGadaaakeaacqWGqbaucqGG7bWEcyGGSbaBcqGGVbWBcqGGNbWzcqWGpbWtcqWGsbGucqGH+aGpcqWGJbWycqGG9bqFcqGH9aqpdaWdraqaaiabdMeajjabcUha7jGbcYgaSjabc+gaVjabcEgaNnaabmaabaWaaSaaaeaacqWGWbaCdaWgaaWcbaGaeGymaeJaeGymaedabeaakiabdchaWnaaBaaaleaacqaIYaGmcqaIYaGmaeqaaaGcbaGaemiCaa3aaSbaaSqaaiabigdaXiabikdaYaqabaGccqWGWbaCdaWgaaWcbaGaeGOmaiJaeGymaedabeaaaaaakiaawIcacaGLPaaaaSqaaiabdchaWnaaBaaameaacqWGPbqAcqWGQbGAcqGHLjYScqaIWaamaeqaaSGaeiilaWYaaabuaeaacqWGWbaCdaWgaaadbaGaemyAaKMaemOAaOgabeaaliabg2da9iabigdaXaadbaGaemyAaKMaemOAaOgabeGdcqGHris5aaWcbeqdcqGHRiI8aOGaeyOpa4Jaem4yamMaeiyFa0Naem4zaCMaeiikaGIaemiCaa3aaSbaaSqaaiabigdaXiabigdaXaqabaGccqGGSaalcqWGWbaCdaWgaaWcbaGaeGymaeJaeGOmaidabeaakiabcYcaSiabdchaWnaaBaaaleaacqaIYaGmcqaIXaqmaeqaaOGaeiilaWIaemiCaa3aaSbaaSqaaiabikdaYiabikdaYaqabaGccqGGPaqkcqqHGoaudaWgaaWcbaGaemyAaKMaemOAaOgabeaakiabdsgaKjabdchaWnaaBaaaleaacqWGPbqAcqWGQbGAaeqaaOGaeiilaWcaaa@88AA@

where *c *is a positive threshold to be chosen from the data and *I*{*A*} represents the indicator function of a set *A*. This can be interpreted as the posterior probability that the log Odds Ratio (logOR) of association between a TF and a miRNA exceeds *c*. Interestingly, if we choose *α *= 0 within our prior distribution, the *p*-value according to Fisher's Exact Test is equal to *P*{log *OR *> 0} [54], implying perfect agreement between the two methods when *c *= 0. We decided to set *α *= 0.001, which practically gives the same results as Fisher's Exact Test (again when *c *= 0), but guarantees that our posterior distribution will be proper. The choice for *c *is important: choosing a small value of *c *would subject our analyses to sample-size effects, yet choosing a value too large can imply even reasonably large significant associations are ignored.

*Choosing a value of c: *Suppose we look at the 5% most highly ranked miRNA-TF pairs (~2000 pairs). As mentioned earlier, if we use p-values from Fisher's Exact Test to create our ranking, most of the TF-miRNA pairs in our list show a large number of targets for both the miRNA and the TF. More explicitly, if we define *a*^(0) ^= (*a*_*l*,*l*_^(0)^, *a*_*l*,*g*_^(0)^, *a*_*g*,*l*_^(0)^, *a*_*g*,*g*_^(0)^) = (0.006, 0.016, 0.255, 0.722) where, for example, *a*_*l*,*g*_^(0) ^is the proportion of the top 2000 pairs for which the number of TF targets is less than 376 and the number of miRNA targets is greater than 253 (note that 376 and 253 are the median number of targets for the respective sets of all TFs and all miRNAs), we see that ranking by Fisher's Exact Test can produce too many pairs where both the TF and miRNA have a large number of targets. We can define a similar vector: *a*^(*c*) ^= (*a*_*l*,*l*_^(*c*)^, *a*_*l*,*g*_^(*c*)^, *a*_*g*,*l*_^(*c*)^, *a*_*g*,*g*_^(*c*)^) for other thresholds. We also define: *a*^(*s*) ^= (*a*_*l*,*l*_^(*s*)^, *a*_*l*,*g*_^(*s*)^, *a*_*g*,*l*_^(*s*)^, *a*_*g*,*g*_^(*s*)^) to be the expected vector of corresponding proportions that would result from a 'ranking procedure' that is not subject to such a sample-size bias. Our choice of *c *minimizes the Euclidean Distance between *a*^(*c*) ^and an estimate of *a*^(*s*)^. Our method for estimating *a*^(*s*) ^is motivated by the observation that if one uses the observed log Odds Ratio to rank TF-miRNA associations, there will be no sample-size bias if the variance of the estimated log Odds Ratio does not change over the pairs. More specifically, to estimate *a*^(*s*)^, we first fit a Generalized Additive Model (GAM) [55] to create a 3D surface displaying predicted logORs as a function of the number of targets of the TF and number of targets of the miRNA. Then, for each TF-miRNA pair, we simulate a value of log⁡(N11N22N12N21)
 MathType@MTEF@5@5@+=feaafiart1ev1aaatCvAUfKttLearuWrP9MDH5MBPbIqV92AaeXatLxBI9gBaebbnrfifHhDYfgasaacH8akY=wiFfYdH8Gipec8Eeeu0xXdbba9frFj0=OqFfea0dXdd9vqai=hGuQ8kuc9pgc9s8qqaq=dirpe0xb9q8qiLsFr0=vr0=vr0dc8meaabaqaciaacaGaaeqabaqabeGadaaakeaacyGGSbaBcqGGVbWBcqGGNbWzdaqadaqaamaalaaabaGaemOta40aaSbaaSqaaiabigdaXiabigdaXaqabaGccqWGobGtdaWgaaWcbaGaeGOmaiJaeGOmaidabeaaaOqaaiabd6eaonaaBaaaleaacqaIXaqmcqaIYaGmaeqaaOGaemOta40aaSbaaSqaaiabikdaYiabigdaXaqabaaaaaGccaGLOaGaayzkaaaaaa@3F57@ by adding a residual to the GAM predicted logOR for that pair. The residual for each pair is chosen to be a random draw from the residuals of the GAM. Having simulated a logOR for each pair, we rank the pairs according to their simulated logORs, and store the resulting vector of proportions: a^
 MathType@MTEF@5@5@+=feaafiart1ev1aaatCvAUfKttLearuWrP9MDH5MBPbIqV92AaeXatLxBI9gBaebbnrfifHhDYfgasaacH8akY=wiFfYdH8Gipec8Eeeu0xXdbba9frFj0=OqFfea0dXdd9vqai=hGuQ8kuc9pgc9s8qqaq=dirpe0xb9q8qiLsFr0=vr0=vr0dc8meaabaqaciaacaGaaeqabaqabeGadaaakeaacuWGHbqygaqcaaaa@2E07@_1_^(*s*)^. Repeating this simulation *n *times and recording a^
 MathType@MTEF@5@5@+=feaafiart1ev1aaatCvAUfKttLearuWrP9MDH5MBPbIqV92AaeXatLxBI9gBaebbnrfifHhDYfgasaacH8akY=wiFfYdH8Gipec8Eeeu0xXdbba9frFj0=OqFfea0dXdd9vqai=hGuQ8kuc9pgc9s8qqaq=dirpe0xb9q8qiLsFr0=vr0=vr0dc8meaabaqaciaacaGaaeqabaqabeGadaaakeaacuWGHbqygaqcaaaa@2E07@_*i*_^(*s*) ^on step *i *we use a^(s)=1n∑a⌢i(s)
 MathType@MTEF@5@5@+=feaafiart1ev1aaatCvAUfKttLearuWrP9MDH5MBPbIqV92AaeXatLxBI9gBaebbnrfifHhDYfgasaacH8akY=wiFfYdH8Gipec8Eeeu0xXdbba9frFj0=OqFfea0dXdd9vqai=hGuQ8kuc9pgc9s8qqaq=dirpe0xb9q8qiLsFr0=vr0=vr0dc8meaabaqaciaacaGaaeqabaqabeGadaaakeaacuWGHbqygaqcamaaCaaaleqabaGaeiikaGIaem4CamNaeiykaKcaaOGaeyypa0ZaaSaaaeaacqaIXaqmaeaacqWGUbGBaaWaaabqaeaacuWGHbqygaWeamaaDaaaleaacqWGPbqAaeaacqGGOaakcqWGZbWCcqGGPaqkaaaabeqab0GaeyyeIuoaaaa@3CE1@ as an estimate of *a*^(*s*)^. Using n = 1000, and a coarse grid of *c *values {0, 0.1, 0.2, ..., 1}, the optimal threshold for *c *was found to be between 0.6 and 0.7. We used a value of 0.6 in our analysis.

## Authors' contributions

YZ initiated, implemented the project and drafted the manuscript. JF and JTC proposed and implemented Bayesian method and drafted the manuscript. YK proposed, initiated, directed the project and drafted the manuscript. All authors read and approved the final manuscript.
